# Supplementary material for: Pre-vaccination and early B cell signatures predict antibody response to SARS-CoV-2 mRNA vaccine
Source: medRxiv. 2021 Jul 7:2021.07.06.21259528. Preprint. [Version 1] doi: 10.1101/2021.07.06.21259528 (PMC8282109; doi:10.1101/2021.07.06.21259528)
Supplement: Supplement 1 [file media-1.pdf]

Kardava et al. Supplementary Materials

Extended Data Figures 103

Extended Tables 1-5

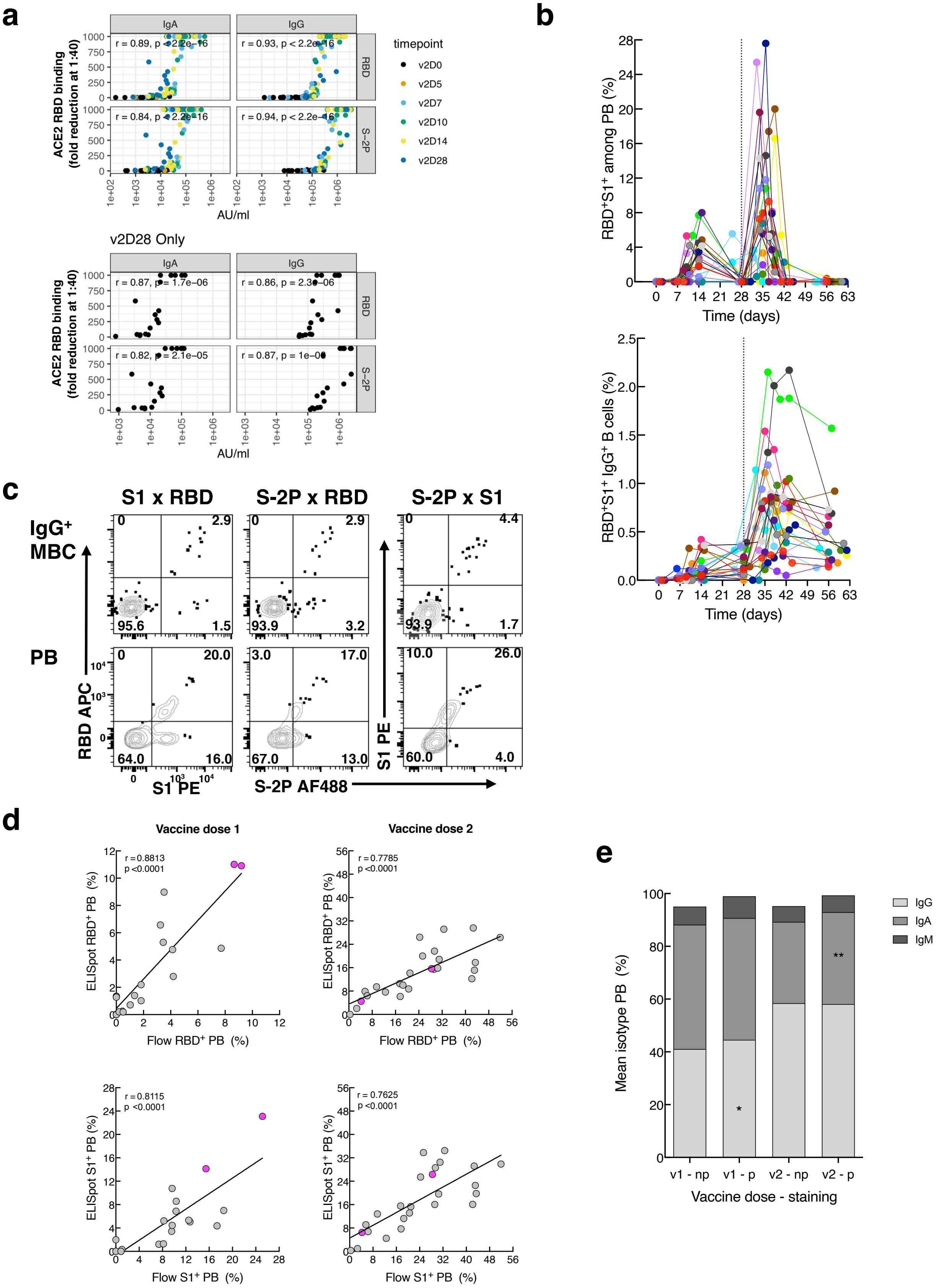
a
IgA
IgG
RBD
S–2P
r = 0.89, p < 2.2e–16
r = 0.93, p < 2.2e–16
r = 0.84, p < 2.2e–16
r = 0.94, p < 2.2e–16
ACE2 RBD binding (fold reduction at 1:40)
AU/ml
timepoint
v2D0
v2D5
v2D7
v2D10
v2D14
v2D28
v2D28 Only
r = 0.87, p = 1.7e–06
r = 0.86, p = 2.3e–06
r = 0.82, p = 2.1e–05
r = 0.87, p = 1e–06
b
RBD+S1+ among PB (%)
Time (days)
RBD+S1+ IgG+ B cells (%)
c
S1 x RBD
S-2P x RBD
S-2P x S1
IgG+ MBC
PB
RBD APC
S1 PE
S-2P AF488
d
Vaccine dose 1
Vaccine dose 2
r = 0.8813
p <0.0001
r = 0.7785
p <0.0001
r = 0.8115
p <0.0001
r = 0.7625
p <0.0001
ELISpot RBD+ PB (%)
Flow RBD+ PB (%)
ELISpot S1+ PB (%)
Flow S1+ PB (%)
e
Mean isotype PB (%)
IgG
IgA
IgM
v1 - np
v1 - p
v2 - np
v2 - p
Vaccine dose - staining

Extended Data Fig. 1. Antibody and cellular assays with validations and correlations. a, Correlations between serum IgG and IgA binding to RBD (Fig. 1b) and ACE2 inhibition assay (Fig. 1e) with v2 timepoints shown with color-coding (top panel) or exclusively measurements at v2D28 (lower panel). **b,** Longitudinal frequencies of $RBD^{+}S1^{+}$ PB and IgG^{+} B cells vaccinees (*n* = 21) color-coded as in Fig. 1. **c,** Binding by flow cytometry of S-2P, S1 and RBD tetramers to PB and IgG^{+} B cells of an individual at v2D6. **d,** Correlation between flow cytometric and ELISpot assays measuring frequencies of RBD^{+} and $S1^{+}$ PB at peak dose 1 and 2 post mRNA-1273 vaccination. **e,** Average PB isotype distribution measured by flow cytometry in presence or absence of permeabilization for donors/dose timepoints in **(d)**. Donors **(d,e)** were participants in protocol NCT00001281 (n = 20) and NCT04411147 (n = 6). Of the 26 donors, two had prior SARS-CoV-2 infection (magenta circles in **(d)**). Spearman's rank correlation **(a,d)**. Paired t test.*, $p < 0.05$; **, $p < 0.01$ **(e)**. AU, arbitrary units; D, day; NP, not permeabilized; P, permeabilized; PB, plasmablasts; RBD, receptor binding domain; S1, spike subunit 1; S-2P, stabilized spike trimer; v, vaccine dose; V, vaccinees.

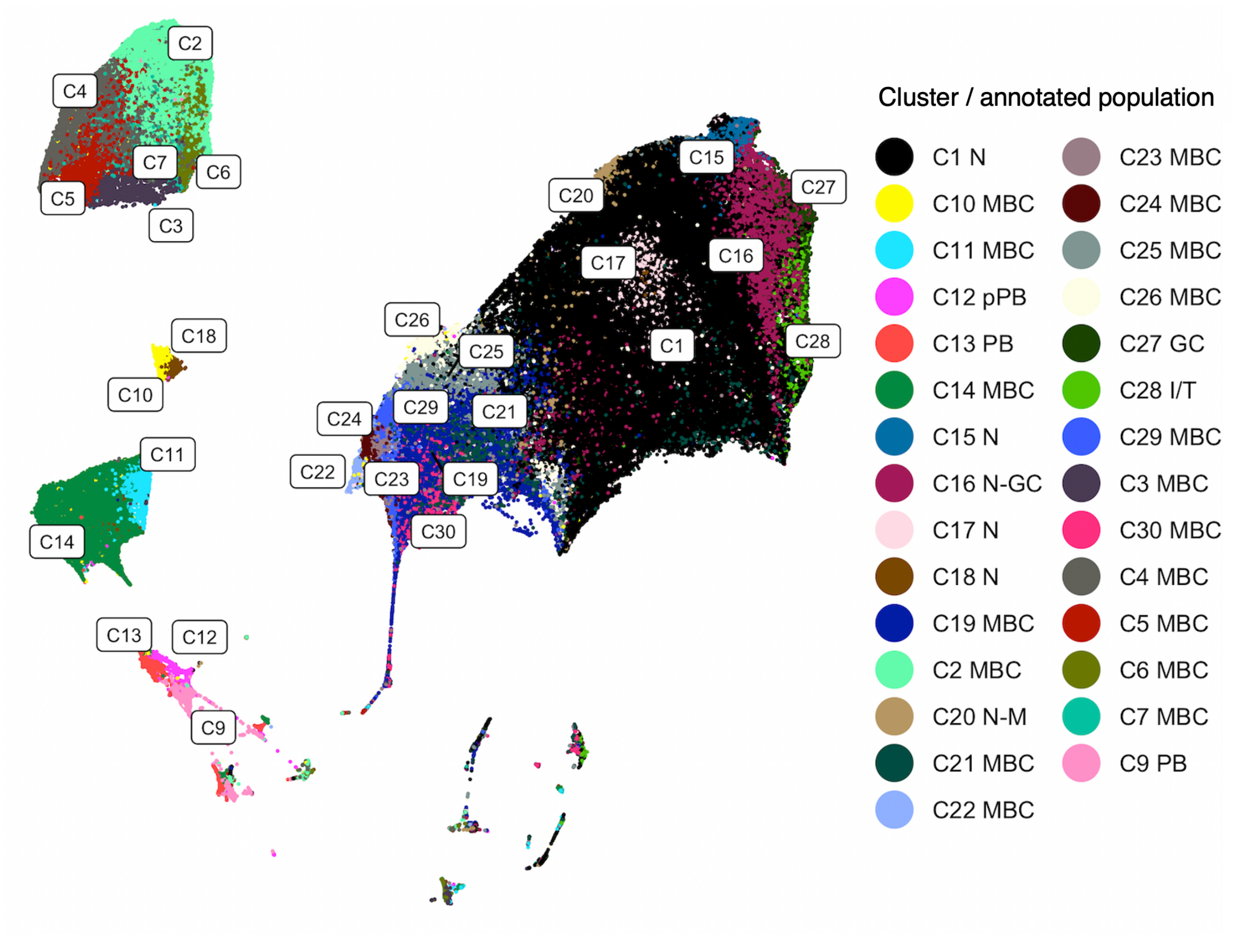

Extended Data Fig. 2. Uniform Manifold Approximation and Projection (UMAP) displaying location of individual cell population clusters. Similar to Fig. 2a but displaying annotations of individual clusters.

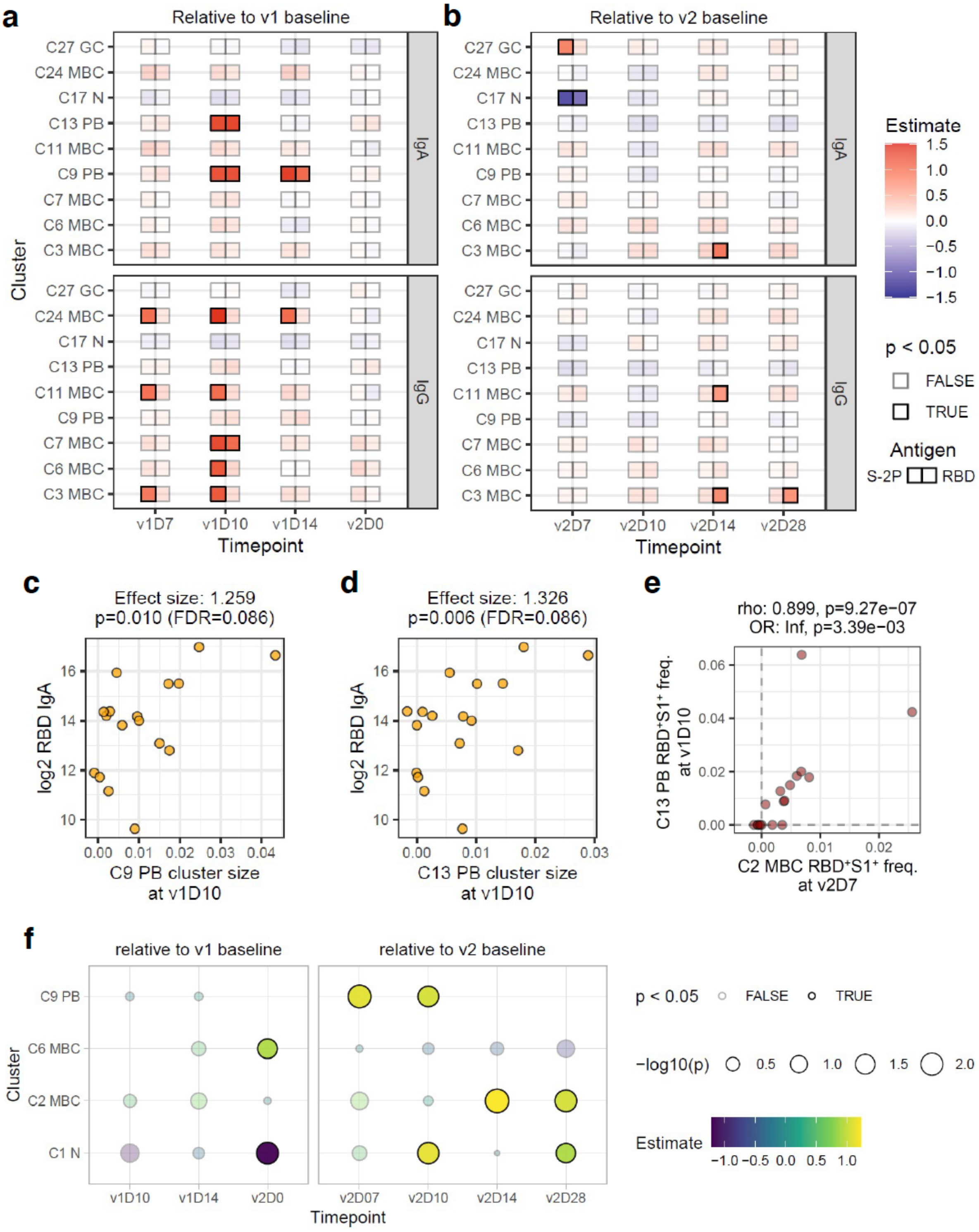
a
Relative to v1 baseline
b
Relative to v2 baseline
C27 GC
C24 MBC
C17 N
C13 PB
C11 MBC
C9 PB
C7 MBC
C6 MBC
C3 MBC
Cluster
IgA
IgG
v1D7
v1D10
v1D14
v2D0
v2D7
v2D10
v2D14
v2D28
Timepoint
Estimate
1.5
1.0
0.5
0.0
−0.5
−1.0
−1.5
p < 0.05
FALSE
TRUE
Antigen
S-2P
RBD
c
Effect size: 1.259
p=0.010 (FDR=0.086)
log2 RBD IgA
C9 PB cluster size
at v1D10
d
Effect size: 1.326
p=0.006 (FDR=0.086)
C13 PB cluster size
at v1D10
e
rho: 0.899, p=9.27e−07
OR: Inf, p=3.39e−03
C13 PB RBD⁺S1⁺ freq.
at v1D10
C2 MBC RBD⁺S1⁺ freq.
at v2D7
f
relative to v1 baseline
relative to v2 baseline
C9 PB
C6 MBC
C2 MBC
C1 N
v1D10
v1D14
v2D0
v2D07
v2D10
v2D14
v2D28
Timepoint
−log10(p)
0.5
1.0
1.5
2.0

Extended Data Fig. 3. Additional associations between cluster frequencies and endpoint SARS-CoV-2 antibody titers. a, b, Similar to Fig. 4a and b, but for associations with antigen non-specific cells. **c, d,** Similar to Fig. 4c, but showing association between endpoint RBD IgA titers and antigen non-specific cells, i.e., cluster size as a fraction of total $CD19^+$ cell counts, within C9 **(c)**, and C13 **(d)**, on v1D10. **e,** Correlation between RBD^+S1^+ cell frequencies within C2 on v2D7 and C13 on v1D10. Spearman's correlation coefficients (rho) and unadjusted P values shown. **f,** Similar to Fig. 4f, but showing association between the first principal component (PC1) of endpoint SARS-CoV-2 antibody titers and spike-specific RBD^+S1^+ (double positive) cell frequencies within each cell cluster. D, day; FDR, false discovery rate; OR, odds ratio; RBD, receptor binding domain; S1, spike subunit 1; v, vaccine dose.

Extended Table 1: Participant information and visits

Participant	Age Range at Vaccination	Gender at Birth	Vaccine Dose 1[#]				Vaccine Dose 2[#]						Days Between Doses
			D0	D7	D10	D14	D0	D5	D7	D10	D14	D28	
VAC-001	51-55	M	1	7	9	14	0		6	9	13	28	28
VAC-002	56-60	F	0	7	11	14	0		7	9	14	28	27
VAC-003	61-65	F	0	6	9	14	0		6	9	15	28	28
VAC-004	51-55	F	0	7	9	15	-1		6	9	13	28	28
VAC-005	61-65	F	0	7	11	14	0		6	11	14	33	28
VAC-611	51-55	F	-21	7	10	14	0		7	9	14	28	29
VAC-613	56-60	F	2	7	10	15	0		7	10	13	29	28
VAC-638	56-60	F	0	7	10	14	0	5	7		14	31	31
VAC-662	51-55	M	0			15	0	5	7	9	14	28	28
VAC-676	51-55	M	0	8		15	-1		6	10	14	31	29
VAC-683	61-65	M	0	7	10	15	0		7	9	14	28	30
VAC-685	41-45	M	-1	7	10	14	0		7	10	14	28	28
VAC-687	51-55	F	0	8	11	14	0		7	11	14	28	28
VAC-713*	41-45	M	1	6		14							
VAC-715	41-45	F	0	7			0		7	10	14	28	25
VAC-716	41-45	F	0	7	12	14	0		7	11	14	28	29
VAC-717	56-60	F		7	11	14	0	5	7	9		28	34
VAC-718	36-40	F	-1	7	11		0		7	11	13	31	28
VAC-719	56-60	M		7	11	14	0		7	11	14	28	28
VAC-720	36-40	M	0	7	10	14	0		6	11	14	28	27
VAC-721	61-65	M		7	10	15	-1		6	10	12	26	26
**Median[†]/N**	**55[†]**	**9M/11F**	**18**	**20**	**17**	**19**	**20**	**3**	**20**	**19**	**19**	**28**	**28[†]**

[*]Lost to follow-up after contracting COVID-19 after dose 1
[#]Numbers indicate actual date of visit relative to vaccination day

Extended Data Table 2: 17-color flow cytometry panel

Reagent	Clone	Source	Catalogue Number	Dilution
Mouse anti-human CD11c BUV395	B-ly6	BD Biosciences	563787	1:100
Mouse anti-human CD138 BUV737	MI15	BD Biosciences	612834	1:200
Mouse anti-human CD45 BUV805	HI30	BD Biosciences	612891	1:100
Mouse anti-human CD38 BV421	HIT2	BD Biosciences	562444	1:100
Mouse anti-human CD19 BV650	SJ25-C1	BD Biosciences	563226	1:100
Mouse anti-human CD10 BV510	HI10a	BD Biosciences	563032	1:100
Mouse anti-human CD3 BV570	UCHT1	Biolegend	300436	1:100
Mouse anti-human IgD BV605	IA6–2	Biolegend	348232	1:100
Mouse anti-human IgM BV711	MHM-88	Biolegend	314540	1:100
Mouse anti-human CD14 BV750	63D3	Biolegend	367136	1:100
Mouse anti-human CD27 BV785	O323	Biolegend	302832	1:100
Mouse anti-human CD21 FITC	BU32	Biolegend	354910	1:100
Mouse anti-human IgG PE-Cy7	G18–145	BD Biosciences	561298	1:100
Mouse anti-human CD20 APC-H7	2H7	BD Biosciences	560734	1:100
SARS-CoV-2 S protein S1		Biolegend	793806	
SARS-CoV-2 S protein RBD		In-house		

Extended Data Table 3: Detailed cluster annotations and designations

Cluster	Population	Ig isotype	Defining markers	Designations/other names	References
28	I/T	IgM>D	$CD10^{+}CD38^{++}CD27^{-}CD138^{+}$		
16	N-GC	IgD>M	$CD10^{+}CD38^{+}CD27^{lo}$		
27	GC	IgM>D	$CD10^{+}CD38^{++}CD27^{+}CD138^{lo}$	GC founder	
23	MBC	IgD>M	$CD27^{lo}CD38^{-}CD20^{++}CD21^{lo}CD11c^{lo}$	Nonconventional MBC/Atypical/TLM	37,38
29	MBC	IgM>D	$CD27^{+}CD38^{-}CD20^{++}CD21^{lo}CD11c^{lo}$	Nonconventional MBC/Atypical/AM/ABC	24,37,38
30	MBC	IgM>D	$CD27^{+}CD38^{-}CD138^{+}$	Conventional MBC	
21	N	IgD=M	$CD38^{-}$		
19	MBC	IgM>D	$CD27^{+}CD38^{-}$	Conventional MBC	
25	MBC	IgM>D	$CD27^{+}CD38^{+}$	Conventional MBC	
22	MBC	$IgD^{+}IgM^{-}$	$CD27^{+}CD38^{-}$	Conventional MBC/IgD only MBC	
24	MBC	IgD>M	$CD27^{lo}CD38^{-}CD20^{++}CD21^{lo}CD11c^{+}$	Nonconventional MBC/Atypical/TLM	37,38
15	MBC	IgD>M	$CD27^{lo}CD38^{+}CD11c^{+}$	Nonconventional MBC	
20	N-MBC	IgD>M; IgG	$CD27^{lo}CD38^{+}$	Likely bound IgG	
1	N	IgD>M	$CD38^{+}$		
17	N	$IgD^{+}IgM^{-}$	$CD38^{+}$		
12	pPB	IgA	$CD27^{lo}CD38^{++}CD20^{-}CD21^{lo}$		
13	PB	IgA	$CD27^{+}CD38^{+++}CD20^{-}CD21^{lo}$		
9	PB	IgG	$CD27^{+}CD38^{+++}CD20^{-}CD21^{lo}$		
8	NB	Multiple Ig	$CD10^{+}CD14^{lo}$	Granulocyte	
5	MBC	IgG	$CD27^{+}CD38^{-}CD20^{++}CD21^{lo}CD11c^{lo}$	Nonconventional MBC/Atypical/AM/ABC	24,37,38
3	MBC	IgG	$CD27^{-}CD38^{-}CD20^{++}CD21^{lo}CD11c^{+}$	Nonconventional MBC/Atypical/TLM/DN2	37-39
4	MBC	IgG	$CD27^{+}CD38^{-}$	Conventional MBC	
2	MBC	IgG	$CD27^{+}CD38^{+}$	Conventional MBC	
7	MBC	IgG	$CD27^{-}CD38^{-}$	Nonconventional MBC	
6	MBC	IgG	$CD27^{-}CD38^{+}$	Nonconventional MBC	
11	MBC	IgA	$CD27^{lo}CD38^{-}CD20^{++}CD21^{-}CD11c^{+}$	Nonconventional MBC/Atypical/TLM/DN2	37-39
14	MBC	IgA	$CD27^{+}CD38^{+}$	Conventional MBC	
26	MBC	$IgM^{+}IgD^{-}$	$CD27^{+}CD38^{+}$	Conventional MBC/IgM only memory	
18	N	Low multiple Ig	$CD38^{+}$		
10	MBC	Low multiple Ig	$CD27^{+}CD38^{lo}$	Conventional MBC	

ABC, activated B cell; AM, activated memory; DN2 double negative 2; GC, germinal center; I/T immature/transitional; MBC, memory B cell; N, naïve; NB, not B cell; PB, plasmablast; pPB, pre-PB; TLM, tissue-like memory

Extended Data Table 4: COVID-19 patient information

Patient	Age Range at enrollment	Gender at birth	Disease day* of sample
4001	36-40	M	20
4004	21-25	M	34
4005	26-30	F	35
4007	61-65	M	19
4008	51-55	F	27
4009	56-60	M	24
4301	66-70	M	39
4302	41-45	F	29
4303	36-40	M	23
002	71-75	M	73
003	36-40	M	35
004	26-30	M	36
005	26-30	F	38
007	81-85	M	32
008	56-60	F	35
010	51-55	M	70
011	46-50	M	17
012	71-75	M	34
013	56-60	M	37
014	46-50	F	18
015	41-45	M	15
**Median†/N**	**48†**	**15M/6F**	**34†**

*At peak antibody response or latest timepoint available

54 **Extended Data Table 5: Additional reagents for flow cytometric analyses**

55

Reagent	Clone	Source	Catalogue Number	Dilution
Mouse anti-human CD3 BV510	OKT3	Biolegend	317332	1:100
Mouse anti-human CD27 BV421	O323	Biolegend	302824	1:100
Mouse anti-human IgD PE-Cy7	IA6-2	Biolegend	348210	1:100
Mouse anti-human IgM APC	MHM-88	Biolegend	314510	1:200
Mouse anti-human IgG PE	G18–145	BD Biosciences	555787	1:40
Mouse anti-human IgA FITC	IS11-8E10	Miltenyi Biotec	130-113-475	1:200
Mouse anti-human CD19 PerCP-Cy5.5	SJ25-C1	ThermoFisher	45-0198-42	1:100
Streptavidin-PE		ThermoFisher	S21388	
Streptavidin-APC		ThermoFisher	S32362	
Streptavidin-Alexa Fluor 488		ThermoFisher	S32354	
SARS-CoV-2 S2 super stable trimer		AcroBiosystems	SPN-C82E9	

56
